# Quality Assessment of Single-Channel EEG for Wearable Devices

**DOI:** 10.3390/s19030601

**Published:** 2019-01-31

**Authors:** Fanny Grosselin, Xavier Navarro-Sune, Alessia Vozzi, Katerina Pandremmenou, Fabrizio De Vico Fallani, Yohan Attal, Mario Chavez

**Affiliations:** 1 Sorbonne Université, UPMC Univ. Paris 06, INSERM U-1127, CNRS UMR-7225, Institut du Cerveau et de la Moelle Épinière (ICM), Groupe Hospitalier Pitié Salpêtrière-Charles Foix, 75013 Paris, France; 2 myBrainTechnologies, 75010 Paris, France; xavier.navarro@mybraintech.com (X.N.-S.); alessia.vozzi@gmail.com (A.V.); katerina.pandremmenou@mybraintech.com (K.P.); yohan.attal@mybraintech.com (Y.A.); 3 INRIA, Aramis Project-Team, F-75013 Paris, France; fabrizio.devicofallani@gmail.com; 4 CNRS UMR-7225, Groupe Hospitalier Pitié-Salpêtrière-Charles Foix, 75013 Paris, France; neurodynamicslab@gmail.com

**Keywords:** electroencephalography (EEG), single-channel EEG, muscular artefacts, quality assessment, artefact detection, wearable systems

## Abstract

The recent embedding of electroencephalographic (EEG) electrodes in wearable devices raises the problem of the quality of the data recorded in such uncontrolled environments. These recordings are often obtained with dry single-channel EEG devices, and may be contaminated by many sources of noise which can compromise the detection and characterization of the brain state studied. In this paper, we propose a classification-based approach to effectively quantify artefact contamination in EEG segments, and discriminate muscular artefacts. The performance of our method were assessed on different databases containing either artificially contaminated or real artefacts recorded with different type of sensors, including wet and dry EEG electrodes. Furthermore, the quality of unlabelled databases was evaluated. For all the studied databases, the proposed method is able to rapidly assess the quality of the EEG signals with an accuracy higher than 90%. The obtained performance suggests that our approach provide an efficient, fast and automated quality assessment of EEG signals from low-cost wearable devices typically composed of a dry single EEG channel.

## 1. Introduction

Electroencephalography (EEG) is the standard method for measuring the electrical activity of the brain with proven efficacy as a tool for understanding cognitive processes and mental disorders. The recent emergence of embedded EEG technology in low-cost wearable devices allows, in addition, to perform EEG recordings in everyday life conditions. Likewise, it offers the possibility to bring mobile, real-time applications to the consumer such as neurofeedback, mental fatigue measurement, sleep monitoring or stress reduction [1]. Often running in uncontrolled environments, portable devices are more susceptible to be contaminated by the typical sources of noise (both internal such as subject movements, blinks, muscular contraction, or external like electro-magnetic interferences, power line noise, etc.) than standard EEG systems [2]. Hence, a fast and robust quality assessment of EEG recordings is of crucial importance in order to provide reliable data for further analysis.

Common ways to assess the quality of an EEG recording system include the comparisons of signal-to-noise (SNR) ratios, event-related potentials (ERP) and steady-state visually evoked potentials (SSVEP) simultaneously estimated by different recording systems [3]. As the visual inspection, these approaches apply an off-line strategy that evaluates the general quality of EEG recordings. Although there is no single rhythm, feature, or characteristic of an EEG that must be present to consider it normal, it is generally accepted that normality on an EEG is simply the absence of identifiable abnormalities [4,5]. The statistical definition of a “clear” EEG signal can provide some help in setting threshold values to determine the artefact level of an EEG recording [2]. These thresholds are generally based on the amplitude, skewness and kurtosis of the EEG signal [2,6,7]. Amplifier drifts or instrumental artefacts, for instance, are generally accompanied by large mean shifts of the EEG amplitudes [6]. Some artefacts like strong muscle activity have a skewed distribution which can be detected by a kurtosis test [7]. Similar thresholds can also be applied to spectral features to detect instrumental and physiological artefacts on EEG signals [8,9]. On the same idea, authors in [10] proposed a method to assess the skin-sensor contact of wearable EEG sensors in several environments like public parks, offices or in-home. Their approach combines several spectral features to establish a decision rule about the quality of this contact that impacts the EEG quality. Although these threshold-based approaches are commonly used to reject EEG segments, they have two main drawbacks: first, they require to manually define the statistical detection thresholds [7]. Secondly, the specificity and sensitivity of these procedures to distinguish between high and low level of contamination are not straightforward [11].

To address the drawbacks of thresholding, classification-based approaches have been proposed to automatically adapt the decision rule to detect the artefact contamination level [12,13,14]. In [15], authors combine EEG and gyroscope signals with support vector machines (SVMs) to detect head movement artefacts. In [16], a fuzzy-c means clustering method is applied on measurements of the fluctuations of the second-order power amplitudes to determine the quality of the EEG signal.

Blind source separation methods like Independent Component Analysis (ICA) can also be used to detect muscle or cardiac artefacts [17] with a visual selection of the component containing the corresponding activity. Appropriate filtering techniques can be applied to physiological recordings (e.g., electrocardiogram or ocular movements), to detect or reduce some artefacts in real time. For instance, motion artefacts can be detected with a gyroscope and subtracted from the raw EEG signal with an appropriate adaptive filter [18]. In the same idea, an approach based on a FIR filter [19,20] can distinguish, on a single EEG channel, ocular artefacts which are detected as irregular spikes. The main disadvantage of these approaches is, however, that they assume that one or more reference channels with the artefacts waveforms are available. For other approaches, like in [21], an ensemble learning approach is used to detect, in an off-line analysis, EEG segments contaminated with muscle artefacts.

Despite the vast number of solutions proposed to reject or reduce artefacts in EEG signals, most of the proposed solutions are applied to classical EEG multi-channel electrode settings. In the context of wearable EEG recording systems with a reduced number of dry electrodes, few methods are capable of distinguishing between “good” EEG quality signals and different type of artefacts.

In this paper, we propose a classifier-based method that combines a spectral comparison technique to assess the quality of EEG and to discriminate muscular artefacts. It has been purposely designed for reduced electrode sets (or single EEG channel configuration) from portable devices used in real-life conditions. We specifically used the Melomind device (myBrain Technologies, Paris, France), a new portable EEG system based on two dry electrodes, to validate the method. The performance of our approach is also validated on standard wet sensors recording subsets: one from Acticap BrainProducts (GmbH, Gilching, Germany) and another from an artefact-free EEG public database [22] contaminated with simulated artefacts of several types (muscular artefacts, blinks, …). Our method is also compared with another algorithm for artefact detection in single-channel EEG systems [2]. Finally, our method is evaluated for the quality assessment of unlabelled EEG databases.

The remainder of the paper is organized as follows: Section 2 describes the databases and the methods used in the proposed approach. Section 3 presents the statistical assessment of our method in terms of accuracy of artefact detection. Finally, we conclude the paper with a discussion in Section 4.

## 2. Materials and Methods

### 2.1. Databases

In this work we consider three levels of artefact contamination:

- Low quality level (LOW-Q): EEG data with a very poor quality, corresponding to a signal saturation, a recording during sensor peeling off, etc.
- Medium quality level (MED-Q): EEG signal contaminated by standard artefacts like muscular activity, eye blinking, head movements, etc. For this level of contamination, the proposed method also discriminates muscular artefacts (MED-MUSC).
- High quality level (HIGH-Q): EEG signals without any type contamination (head movement, eye blinking or muscular artefacts). These EEG signals are considered as “clean”.

In order to validate our method, we first studied two databases containing EEG signals recorded with different EEG sensors on healthy subjects for whom we asked to deliberately generate different type of artefacts. Thirty seconds of EEG data were recorded for each type of artefacts (including eye blinking, head and eye movements, jaw clenching). Very contaminated data (signal saturation and electrode peeling off), was also deliberately produced during 30 s of recording. Finally, 1 min of EEG data was collected during the subjects were asked to be quiet but alert.

The first database (*artBA*) is composed of EEG signals from three subjects recorded by an Acticap BrainProducts (GmbH, Gilching, Germany) system using 32 wet electrodes in the 10–20 International System. Signals were amplified, digitized at 1000 Hz sampling frequency, then down-sampled to 250 Hz and segmented in one second non-overlapping windows. For all recordings, the impedance between the skin and the sensors was below 5 kΩ.

The second database (*artMM*) is composed of EEG signals from 21 subjects recorded by Melomind (myBrain Technologies, Paris, France), a portable and wireless EEG headset equipped with two dry sensors on P3 and P4 positions according to the 10–20 International System. EEG signals were amplified and digitized at 250 Hz and segmented in one second non-overlapped windows, then corrected to remove DC offset and 50 Hz power line interferences by Melomind’s embedded system before being sent via Bluetooth to a mobile device.

We used a third database (*publicDB*) which comes from the BNCI Horizon 2020 European public repository, dataset 13 [22]. It contains motor imagery-related EEG signals from 9 subjects recorded by g.tec GAMMAsys system using 30 wet active electrodes (g.LADYbird) and two g.USBamp biosignal amplifiers (Guger Technolgies, Graz, Austria). Artefact-free EEG segments were selected to build this database contaminated with artificially-generated artefacts.

Finally, two unlabelled databases containing real EEG activity were also collected on 10 subjects in parietal regions (P3 and P4) with a standard system (Acticap BrainProducts, GmbH, Gilching, Germany) and with a low-cost system (Melomind, myBrain Technologies, Paris, France). One dataset contains therefore EEG recordings made with wet standard (*wetRS*) electrodes, whereas the second one contains the data recorded with the dry sensors (*dryRS*). In all these EEG recordings, the subjects were asked to be at rest with closed eyes but in alert condition during 1 min.

According to the declaration of Helsinki, we obtained written informed consent from all the subjects (of the previous described databases) after explanation of the study, which received the approval from the local ethical committee (CPP-IDF-VI, num. 2016-AA00626-45). More details about the composition (the number of EEG segments and type of artefacts) of each database can be found on Table 1.

### 2.2. Overview of the Method

**Figure 1:**
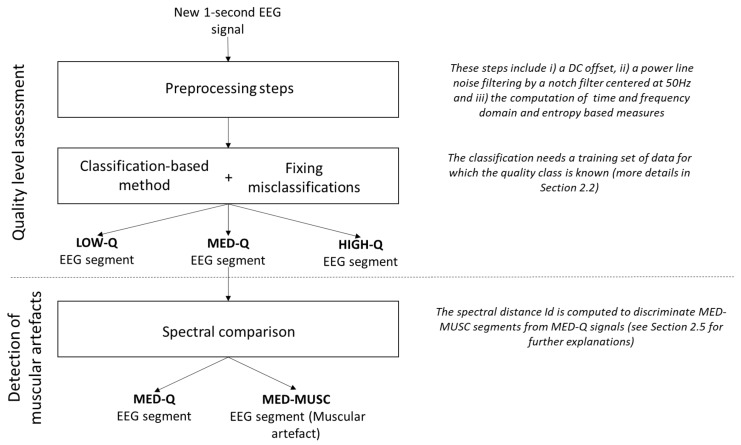
Overview of the contamination level assessment for a single-channel EEG.

Our method, summarized in Figure 1, includes the following main steps:

1. Pre-processing: All EEG recordings are segmented in one second non-overlapping windows. For each segment, the DC offset level is removed and power line noise is suppressed by a notch filter centred at 50 Hz. Then, several time and frequency domain and entropy-based measures are computed (see Section 2.3).
2. Quality assessment: Different classifiers are trained (see Section 2.4 below) on a subset of data (training set), for which the quality class is known, to assign each EEG segment of the remaining subset (testing set) to one of the three levels of artefact contamination (low, medium and high). To reduce the number of misclassifications, EEG segments with more than 70% of constant values (saturation and flat signals) and those with extreme values (±300 μV) are considered as low quality data [6].
3. Discrimination of muscular artefacts: To discriminate muscular artefacts from EEG segments, we compare the spectrum of contaminated segments with a reference spectrum obtained from the training set of clean segments. An EEG segment is considered to include a muscular artefact if the spectral distance exceeds a threshold *T* (details of the method are described in Section 2.5).

### 2.3. Features Extraction

The collection of features used to assess the quality of the EEG segments includes a total of 114 parameters obtained from both time and frequency domains that are commonly used in artefact detection from electrophysiological signals [2,6,7,8,10,23,24], or to detect seizures from neonatal EEG [11,25].

Time-domain features include the maximum value, the standard deviation, the kurtosis and the skewness [7,11,26]. Some of these features were extracted from EEG signals filtered in different frequency bands [2]. In this context, a band-pass filter was applied with specific cut-off frequencies according to the EEG frequency bands: 0.5–4 Hz for δ band, 4–8 Hz for θ band, 8–13 Hz for α band, 13–28 Hz for β band and 28–110 Hz for γ band. See Table A1 (in Appendix A.1) for the full list of time domain features.

Frequency-domain features offer the possibility to quantify changes in the power spectrum. Most of these features were inspired from three studies [10,11,23]. Certain features, originally defined for speech recognition and quality assessment of electromyogram, were adapted for EEG signals. Some parameters (like the log-scale or the relative power spectrum) were extracted directly from the spectrum in the frequency bands used for the time domain features. To see the full list of extracted features in frequency domain, see Table A2 (in Appendix A.2).

Supplementary structural and uncertainty information from EEG segments were extracted using Shannon entropy, spectral entropy, and singular value decomposition entropy [11].

### 2.4. Classification-Based Methods

In this work, we compare several classifiers to categorize EEG segments into the three quality levels (low medium and high), using a 5-fold cross validation. Before the classification, the value of each feature (in every EEG segment) was normalized with the mean and standard deviation obtained from EEG segments contained in the training set. The classifiers evaluated in this work are the following:

*Linear Discriminant Analysis (LDA)* is a standard algorithm that finds a linear decision surface to discriminate the classes [27]. This classifier can be derived from simple probabilistic rules which model the class conditional distribution P(X|l) of an observation *X* for each class *l*. The class of each new EEG segment is predicted by using Bayes’ theorem [28]:

(1) $$P\left(l|X\right)=\frac{P(X|l)P(l)}{P(X)}=\frac{P(X|l)P(l)}{\sum_{k}P\left(X|k\right)P\left(k\right)}$$

where P(l) denotes the class priors estimated from the training set by the proportion of instances of class *l*. Although this classifier is easy to interpret and to implement, its performance is sensitive to outliers. We assign to an EEG segment the class *l* which maximizes the conditional probability and minimizes the misclassification rate [28].

*Support Vector Machines (SVMs)* use a kernel-based transformation to project data into a higher dimensional space. The aim is to find a separating hyper-plane in the space between the two classes [29]. Although it exists an infinity of hyper-planes to discriminate the two classes, SVMs keep the hyper-plane which maximizes the distance between the two classes and minimizes the misclassifications. In our case, a “one-against-one” approach is used to solve our multi-class classification problem. This method builds L(L−1)/2 classifiers where *L* is the number of classes. Each classifier is trained on data from two classes [30]. In this work, SVM with linear kernel function is tested (Linear SVM). SVMs present several advantages: they generally provide good performance with fast computations, over-fitting can be avoided by making use of a regularization parameter and non-linear classifications overcome with the choice of the appropriate kernel. However, the setting of such parameters is not straightforward and improper parametrization may result in low performance.

*K-Nearest Neighbours (kNN)* classifier is a simple nonparametric algorithm widely used for pattern classification [31]. An object is classified by a majority vote of its neighbours, with the object being assigned to the most common class among its *k* nearest neighbours in the training set [31]. A neighbour can be defined using many different notions of distance, the most common being the Euclidean distance between the vector *x* containing the feature values of the tested EEG segment, and the vector *y* containing the feature values of each EEG segment from the training set, which is defined as Equation (2):

(2) $$d_{Euclidean}=\sqrt{\sum_{i=1}^{n}{\left(x_{i}-y_{i}\right)}^{2}},$$

where *n* denotes the number of features computed from each EEG segment.

We evaluated the Euclidean distance kNN (referred here as “Euclidean kNN”) but also a weighted kNN (referred as “Weighted kNN”). In the latter, distances are transformed into weights by a distance weighting function using the squared inverse distance, following this equation:

(3) $$d_{weighted}=\frac{1}{d_{Euclidean}^{2}}$$

By weighting the contribution of each of the *k* neighbours according to Equation (3), closer neighbours are assigned a higher weight in the classification decision. The advantage of this weighting schema lies in making the kNN more global which overcomes some limitations of the kNN [32]. In general, the main advantage of kNN is that it does not need a training phase, it is easy to implement, it learns fast and the results are easy to interpret [32]. However, this algorithm can be computationally expensive and is prone to be biased by the value of *k* [32].

### 2.5. Spectral Distance to Distinguish Muscular Artefacts

As mentioned above, our method can discriminate artefacts of muscular origin. For this purpose, a spectral distance is first estimated between all the clean (high-quality or HIGH-Q) segments of the training set. An EEG segment detected with a medium quality (MED-Q) can be further discriminated as a muscular artefact (MED-MUSC) if the distance of its spectrum (Pxx(f)) to the averaged spectrum of clean segments (Pyy(f)) is higher than a threshold *T*, defined as *N* standard deviations above the mean distance computed between all clean segments. For each database, *N* is chosen iteratively so that the accuracy of detection of MED-MUSC segments is maximum in the training set.

Here, we use the Itakura distance, a statistical distance widely used in spectral analysis [33,34], defined by:

(4) $$dI=\log\left(\sum_{f}\frac{Pxx(f)}{Pyy(f)}\right)-\sum_{f}\log\left(\frac{Pxx(f)}{Pyy(f)}\right)$$

where Pxx(f) and Pyy(f) are the spectra to be compared over the spectra frequency range, set here 0<f<40 Hz as it contains the most relevant information of the EEG.

### 2.6. Validation Procedure

#### 2.6.1. Generation of Artefacts

To test the performance of the different classifiers under study, we generated contaminated data both in a real (*True artefacts*) and in an artificial condition (*Synthetic artefacts*), by controlling the level of contamination with respect to the clean EEG.

*True artefacts*: The databases obtained from the standard EEG system (wet electrodes) and the low-cost device (dry sensors) are composed of three sets, each containing a data collection with different types of artefacts:

1. Clean EEG signals without internal or external artefacts recorded while subjects were instructed to remain quiet but alert during 1 min.
2. EEG signals from subjects instructed to deliberately produce 30 s of different type of artefacts like eye blinking, head and eye movements and muscular artefacts (jaw clenching) at short intervals.
3. Very contaminated EEG data after the subjects with deliberately produced signal saturation or electrode peeling off during 30 s.

These datasets were visually inspected by trained EEG experts. 1 s-EEG segments were manually label as LOW-Q, MED-Q, MED-MUSC and HIGH-Q to constitute the ground truth of the classification.

*Synthetic artefacts*: Artificially contaminated EEG signals were simulated using data from the public database described in Section 2.1. Artefacts were generated in three different ways to dispose of the following patterns:

1. Electrooculogram (EOG) signals were first detected by means of a wavelet thresholding procedure [35]. The residual EEG in the EOG was then extracted so only eye-related activity (blinks, slow vertical and horizontal movements) was kept.
2. Muscular artefacts were generated using random noise band-pass filtered between 20 and 45 Hz with a random length between 0.3–0.7 s (equivalent to those observed in real EEG data) [35].
3. Large movements and electrode clipping were simulated by interpolating successive number of extreme values (3 to 5) with an amplitude between 100 and 400 μV and temporally spaced among 10 to 100 ms.

Clean EEG signals (selected by visual inspection) and simulated artefacts came from different subjects to ensure that all segments of simulated and real EEG data were independent of each other. Synthetic artefacts *v* were superimposed on the clean EEG segments *b* of 1 s of duration as follows: $b^{\mathrm{artefacted}}=b+\lambda v$, where λ represents the contribution of the artefact. For each segment and artefact type, the signal to noise ratio (SNR) was adjusted by changing the parameter λ as follows:

(5) $$\mathrm{SNR}=\frac{\mathrm{RMS}(b)}{\mathrm{RMS}(v)}$$

where RMS(b) corresponds to the root mean squared value of the clean segment, and RMS(v) denotes the root mean squared value of the synthetic artefact. We generated 300 segments with SNRs between 0 and 15 dB: 200 for pattern 1 (100 for slow eye movements and 100 for blinks, that were labelled MED-Q) and 100 for pattern 2 (labelled MED-MUSC). For low quality EEG (LOW-Q), we generated 300 excerpts for pattern 3 with SNRs from −10 to 0 dB.

#### 2.6.2. Measures of Performance

Classification performance was measured in terms of accuracy (percentage of correctly detected artefacts) and area under the Receiver Operating Characteristic (ROC) curves (AUC). Here, we computed one ROC curve for each class selected as positive against the other two classes [36]. To evaluate both measures of performance on the classifiers under test, we applied a 5-fold cross-validation procedure.

The EEG signals may be influenced by subject-related characteristics (e.g., skin, scalp thickness or hair), or by technical and environmental factors during the recorded time (e.g., electromagnetic noise levels and humidity levels). EEG recordings cannot therefore be comparable in their quality between subjects or recording times. We notice, however, that the inter-individual variability of resting state as well as physiological (eye movement and blinks, muscular contamination, …) or environmental artefacts is lower than intra-individual variability across time (days or weeks) [37,38]. The cross-validation procedure tests the reliability of our algorithm by testing data collected in different subjects.

## 3. Results

In this section, we describe the measures of performance to select the best features and classification method.

### 3.1. Tuning of Parameters

#### 3.1.1. Feature Selection

A feature selection method was applied to reduce redundancy and hence to avoid the problems associated with high-dimensional space of features. This procedure not only increases classification performance but also reduces computational time in a real-time context [39,40]. We employed the Fast Correlation-Based Filter (FCBF) [41] technique to keep only the features relevant to a class [41] by measuring the correlation between each feature ($f_{i}$, with i=1,…,F) and each class (l=1,…,L) using symmetrical uncertainty (SU) [42]. It is defined as follows:

(6) $$SU\left(f_{i},l\right)=2\times \frac{IG(f_{i}|l)}{H\left(f_{i}\right)+H\left(l\right)}$$

where $IG(f_{i}|l)$ denotes the information gain of the feature $f_{i}$ given the class *l*; H(l) and $H(f_{i})$ are the entropy of the class *l* and feature $f_{i}$, respectively. The information gain $IG(f_{i}|l)$ can be obtained as $H\left(f_{i}\right)-H\left(f_{i}|l\right)$ where $H(f_{i}|l)$ is the conditional entropy feature $f_{i}$ given the class *l*.

A subset *S* of relevant features are chosen by defining a threshold δ of SU value, such that the features $f_{i}$ included in *S* verify $SU(f_{i},l)\geq \delta$. The FCBF method keeps only non-redundant features. A feature $f_{i}$ is considered as redundant if there exists a feature $f_{j}\left(j\neq i\right)$ such that $SU\left(f_{j},f_{i}\right)\geq SU\left(f_{i},l\right)$ [41].

#### 3.1.2. Choice of the Classifier

**Figure 2:**
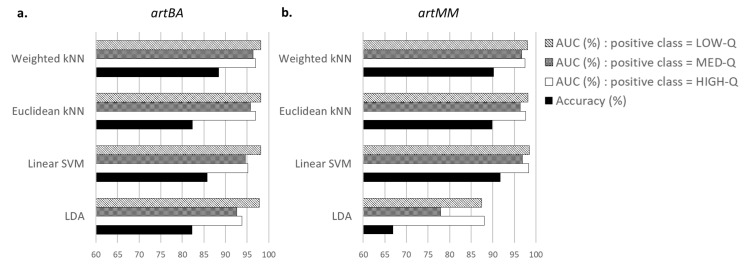
Comparison of classifiers in terms of total accuracy and AUCs (in percentage) of a 5-fold cross validation after features selection on the recordings obtained with (**a**) the standard EEG system (*artBA*) and (**b**) with the dry sensors device (*artMM*). Results are averaged across 5 independent runs.

After selecting the most relevant features, the choice of the classifier was based on the comparison of total accuracy and AUC scores between four classifiers. Figure 2 presents the average values over 5 runs for the recordings acquired with the standard EEG system (Figure 2a) and with the dry electrodes device (Figure 2b) databases. At each run, all the EEG segments of each class were randomly shuffled.

Results from databases with wet EEG electrodes are displayed in Figure 2a. We can observe that the Weighted kNN has the best accuracy (88.38%), followed by the Linear SVM (85.8%). AUC values indicate that differences among classifiers are negligible, with scores reaching 95% except for LDA that performed slightly less.

Classifiers’ performance on signals acquired with dry electrodes are in Figure 2b. Although Weighted kNN accuracy (90.22%) does not provide the highest performance, it is very close to the best value (91.75%) obtained by the Linear SVM. As for wet sensors, AUC scores exceed 95% except for LDA.

To study the impact of the number of nearest neighbors (*k*) used in Weighted kNN classification, we obtained the accuracy values corresponding to *k* from 1 to 20, averaged after applying a 5-fold cross validation on each database. The best result is obtained with k=7 for *artBA* and *artMM*; and k=10 for the artificially contaminated EEG segments from the public database (*publicDB*).

**Figure 3:**
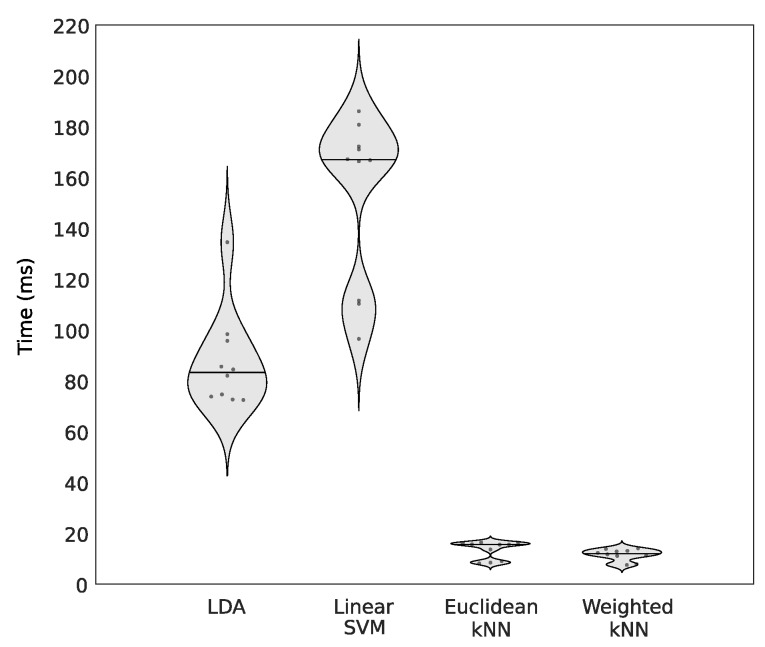
Execution times to predict the quality of 1 s EEG segment for each classifier. The straight line in each violin plot, represents the median value.

A complementary criterion to compare the four classifiers, decisive for wearable devices working in a real-time setting, is the execution time to predict the quality of EEG segments. As it can be observed in Figure 3, the Weighted kNN is the fastest algorithm, providing predictions 12 times faster than the slowest solution, the Linear SVM. It is important to notice that although the Weighted kNN has lower accuracy than the Linear SVM for the EEG recoded with the dry sensors device, it provides faster classifications (computation times divided approximately by 12). Although Linear SVM provides slightly better accuracies than Weighted kNN, the latter is the best trade-off regarding classification performance and computational complexity for a real-time implementation.

#### 3.1.3. Muscular Artefact Detection Settings

The threshold *T* allowing to distinguish muscular artefacts is defined as *N* standard deviations above the mean distance computed between all clean segments of the training set. *N* is chosen so that a maximum number of muscular artefacts can be separated from the other EEG segments containing artefacts in the training set, avoiding false positive and negative assignments. Following this procedure, *N* is set to 8 for *artBA*. For *artMM* and *publicDB*, *N* is set to 2.5 and 0.5 respectively.

### 3.2. Assessment of Quality Checker’s Performances

The performance of our quality checker was evaluated on each database for which the ground truth is known (*artBA*, *artMM* and *publicDB*), by the method described in Section 2.2. The total accuracy and the accuracies in each class, are then computed (see Table 2).

Table 2 shows that the proposed method successfully classifies more than 90% of EEG segments in each database. The highest accuracies concerned LOW-Q segments, with 94.11% for *artBA*, 96.67% for *artMM* and 99.67% for *publicDB*. Right after, HIGH-Q EEG is successfully detected in 92.11% of the segments for *artBA*, 91.05% for *artMM* and 95.67% for *publicDB*. Finally, artefact detection in EEG segments with moderate artefacts provides accuracy values of 87.11% for *artBA*, 84.86% for *artMM* and 88.87% for *publicDB*. Concerning the muscular artefacts, 94.4% (for *artBA*), 91.2% (for *artMM*) and 86.02% (for *publicDB*) of the EEG segments classified as MED-Q, are correctly detected as MED-MUSC.

### 3.3. Comparison with Another Artefact Detector

We have compared our results with the performance obtained with another semi-automatic method for identifying artefacts in single-channel EEG [2]. Briefly, this algorithm determines if a given portion of EEG falls within the thresholds of clean EEG. The metrics used for this comparison include the maximum, the standard deviation, the kurtosis and the skewness of the amplitude extracted from raw and filtered (in the frequency bands of 8–12 Hz and 13–35 Hz) signals. The value of each metric is then checked against a set of threshold values (one threshold per metric). It is worthy to note that this method only discriminates between two classes: clean (HIGH-Q) and contaminated (LOW-Q and MED-Q) EEG segments. Results are on Table 3.

When compared with the results from Table 2, we can observe that the performance of the threshold-based method is lower than those obtained with our classifier-based method. The good detection performance of low quality segments in *publicDB* database can be explained by the construction of this artificial contaminated data. Indeed, these EEG segments contain some extreme values whose amplitudes are higher than the fixed thresholds. The poor detection of the contaminated segments can be due to the features used by the algorithm, which probably cannot completely characterize the different artefacts, thus unable to discriminate them from clean EEG signal. These results can also be explained by the use of fixed thresholds. Indeed, although identified thresholds encompass the majority of the maxima extracted from the clean EEG, they do not include them all.

### 3.4. Quality Assessment of Unlabelled EEG Recordings

To evaluate our algorithm in real unlabelled EEG recordings, two databases were collected on 10 subjects during a resting state condition with a standard EEG system (Acticap BrainProducts, GmbH, Gilching, Germany) and with a low-cost system (Melomind, myBrain Technologies, Paris, France). To determine the quality level of each segment in the new (unlabelled) databases, the classifiers were trained on the EEG signals contained in the labelled databases (see Section 2.1). For the quality assessment of EEG segments, weighted kNN classifiers were used, with *k* set to 7 as previously used (see Section 3.1.2). The percentage of detected artefacts are indicated on Table 4.

Results show that most of the recordings in both databases are detected as clean EEG data (HIGH-Q): 91.50% for the standard EEG setting and 80.58% for the dry electrodes device. Only a few segments are detected as LOW-Q quality level: 1.25% of EEG acquired with wet electrodes and 0.9% of signals recorded with dry sensors. Finally, 7.25% and 18.50% of segments are detected with MED-Q quality level for the standard EEG headset and mobile device, respectively.

The assessment of quality suggests that both datasets are practically free of artefacts, as expected when subjects were asked to be at rest with eyes closed. The slightly higher proportion of contaminated segments detected in the recordings of the low-cost device can be explained by the fact that the contact between dry sensors and the skin is poorer than that obtained by the wet sensors of the standard EEG system.

### 3.5. Impact of the Contamination Level

**Figure 4:**
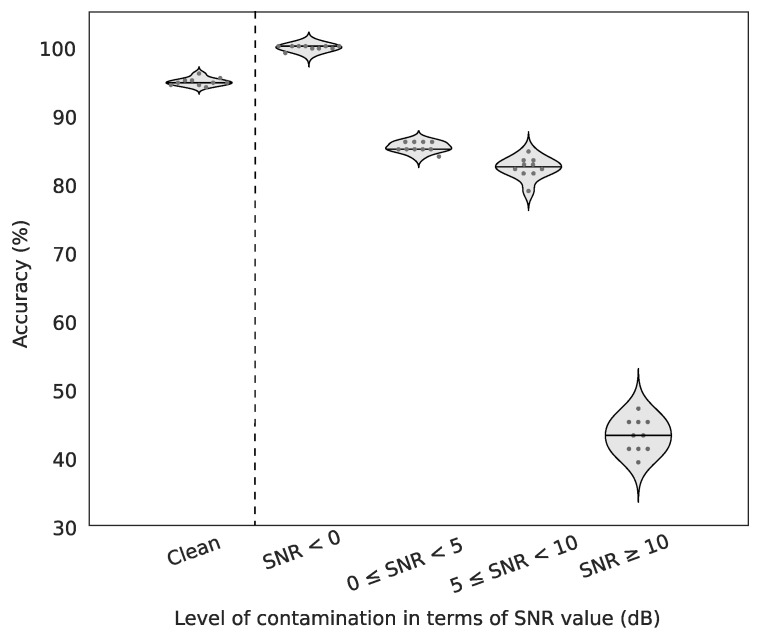
Accuracy of EEG quality checker for different levels of contamination. The accuracy of detection is assessed on no contaminated data (referred as “Clean”) and for different levels of contaminated data. The level of contamination is described by the SNR value as explained in Section 2.6.1. Ten independent runs were performed to compute the accuracies of detection. Each run was done with a 5-fold cross validation. The straight line in each violin plot represents the median value.

As introduced in Section 2.1, the public database served to generate artefacted EEG by controlling the degree of contamination (artefacts) and hence to evaluate the sensitivity of our method in different SNRs defined earlier (see Figure 4). We therefore used Weighted kNN with k=10 and repeated a 5-fold cross-validation procedure 10 times, each run with randomly selected training set. In this database, the averaged percentage of correctly detected clean segments is equal to 94.8% whereas the most contaminated excerpts (SNR < 0 dB) reach in almost perfect detections (99.8% mean accuracy). For moderate artefacts (0 ≤ SNR < 10 dB) correct predictions range from 80 to 85%, a good result if we consider that SNRs between 5 and 10 dB are hardly recognizable visually. Finally, in the most challenging scenario (SNR ≥ 10 dB) performance drastically decreases (43.13% mean accuracy) because of the low level of added artefacts. As the misclassified segments are mostly labelled as HIGH-Q, the eventual impact on subsequent EEG analysis is negligible.

### 3.6. Execution Time

The execution time of the proposed method was computed and averaged through 10 runs, using a 2.5 GHz dual-core Intel Core i5 processor, of 8 GB memory. Using Matlab (version R2017b) our approach estimates the quality of 1 s EEG segment in 14.3 ms on average. In comparison, a classification using Linear SVM is ten times slower than the proposed approach. Our algorithm was also implemented in C++ language to be used in embedded EEG systems, and it takes, on average, 3.2 ms to assess the quality of 1 s EEG data.

## 4. Discussion and Conclusions

The proposed approach is a classification-based method to evaluate the quality of EEG data that includes a spectral distance to discriminate muscular artefacts from the other types of artefacts. We propose a fast and efficient an approach for detecting and characterizing artefacts that can be applied in single-channel EEG configurations. The method was validated on different databases containing real artefacts generated in real conditions, and one database with artificially generated artefacts superimposed to clean EEG data.

A comparison of performance in terms of accuracy and AUC was made to choose the best classifier among the LDA, Linear SVM, Euclidean and Weighted kNNs. Although the Linear SVM obtained slightly better accuracies for one of the tested databases, the Weighted kNN was selected as a good compromise regarding the artefact detection and the execution time. Indeed, for each labelled database (for which the quality level of segments was known), the proposed approach with a Weighted kNN reached more than 90% of good detection in the quality assessment of EEG segments, taking less than 15 ms for each EEG segment.

For artificially contaminated EEG signals, we show that our method may yield almost perfect detections in moderate to high artefactual conditions and very fair performance even with high signal-to-noise ratios. Indeed, contaminated signals with SNR between 0 and 10 dB were detected more than 80% of times although they are hardly recognizable visually. Currently, the algorithm can detect muscular artefacts but further investigations will be performed to automatically recognize specific patterns of other sources of artefact contamination (blinks, saccades, head movements, …).

When applied to the unlabelled databases, our algorithm detected similar amount of contaminated segments on EEG recordings from both the standard EEG system (with wet electrodes) and the dry sensor device. These results were in full agreement with the high quality of EEG recordings obtained during resting state, where the subjects were asked to be at rest with eyes closed.

Finally, the results presented in this work suggest that our approach is a good EEG quality checker in off-line environments with either dry or wet EEG electrodes. The presented algorithm is not subject-driven and classifiers are trained with data collected from different subjects at different time periods. Although beyond the scope of our study, we notice that an optimization of subject-driven classifiers for longitudinal recordings (weeks or months) might increase classification performance. Results indicate that the proposed method is suitable for real-time applications dealing with embedded EEG in mobile environments, such as the monitoring of cognitive or emotional states, ambulatory healthcare systems [43] or sleep stage scoring. In practice, this method is currently used to provide an efficient, fast and automated quality assessment of EEG signals recorded in uncontrolled environments with Melomind (myBrain Technologies, Paris, France), a low-cost wearable device composed of two dry EEG channels.

## 5. Patents

The proposed approach reported in this manuscript is part of a patent application, with date of 29 June 2018, entitled “Multiclass classification method for the estimation of EEG signal quality”, submitted by myBrain Technologies, F.G., X.N.-S. and Y.A.

## Figures and Tables

**Table 1:** Composition of each database in terms of number of LOW-Q, MED-Q, MED-MUSC, HIGH-Q labelled EEG segments.

	LOW-Q	MED-Q (MED-MUSC)	HIGH-Q	TOTAL
*artBA*	98	98 (18)	98	294
*artMM*	210	210 (45)	210	630
*publicDB*	300	300 (100)	300	900
*wetRS*	-	-	-	1200
*dryRS*	-	-	-	1200

**Table 2:** Detection accuracy values obtained for each class of contaminated segments (LOW-Q, MED-Q and HIGH-Q) in the three datasets (*artBA*, *artMM*, *publicDB*). For MED-MUSC segments, accuracy is computed on EEG segments classified as MED-Q by the Weighted kNN.

	LOW-Q	MED-Q (MED-MUSC)	HIGH-Q	TOTAL
*artBA*	94.11%	87.11% (94.4%)	92.11%	91.09%
*artMM*	96.67%	84.86% (91.2%)	91.05%	90.86%
*publicDB*	99.67%	88.87% (86.02%)	95.67%	94.73%

**Table 3:** Detection accuracy values obtained from the threshold-based method for each class of contaminated segments (LOW-Q, MED-Q and HIGH-Q) in the labelled databases.

	LOW-Q	MED-Q	HIGH-Q	TOTAL
*artBA*	54.08%	68.37%	72.48%	64.97%
*artMM*	84.29%	64.76%	82.38%	77.14%
*publicDB*	100%	38%	76.67%	71.56%

**Table 4:** Quality detection with the proposed method in databases composed of EEG segments collected during a resting state task (*wetRS* and *dryRS* denote the databases acquired with the standard wet EEG electrodes, and dry EEG sensors, respectively). The values indicate the percentage of detected segments in each class (LOW-Q, MED-Q and HIGH-Q).

	LOW-Q	MED-Q	HIGH-Q
*wetRS*	1.25%	7.25%	91.50%
*dryRS*	0.9%	18.50%	80.58%

## Abbreviations

The following abbreviations are used in this manuscript:

AUC	Area Under the Curve
ECG	Electrocardiogram
EEG	Electroencephalography
EOG	Electrooculogram
ERP	Event-Related Potentials
FCBF	Fast Correlation-Based Filter
FFT	Fast Fourier Transform
H	Entropy
HIGH-Q	High quality level
IG	Information Gain
kNN	k-nearest neighbour
LDA	Linear Discriminant Analysis
LOW-Q	Low quality level
MED-MUSC	Muscular contamination
MED-Q	Medium quality level
ROC	Receiver Operating Characteristic
SNR	Signal-to-Noise
SSVEP	Steady-State Visually Evoked Potentials
SU	Symmetrical Uncertainty
SVM	Support Vector Machine

## Appendix A

### Appendix A.1. Time Domain Features

The full list of features, extracted from the time domain [11,26], is listed in Table A1, and are summarized as follows:

The root mean square amplitude and the difference between the highest and the lowest value are directly based on the extreme values of the amplitude of the raw EEG signal. The number of local maxima and minima is calculated by summing the number of times that the 1st derivative of the EEG signal is smaller than a specific threshold. The zero-crossing rate is the sum of all positive zero crossings into the EEG segment [11,24]. This feature can be also computed for the 1st and the 2nd derivative of the signal. Some measures characterize the EEG amplitude distribution [6,7] like the mean, the median, the variance, the maximum, the skewness, the kurtosis, the 2nd and the 3rd Hjorth parameters. The variance is also extracted from the 1st and the 2nd derivative of the EEG signal. Information about changes in amplitude through time is obtained by the average amplitude change between two consecutive data points, the difference absolute standard deviation value and the non-linear energy usually used to detect spikes. The integrated EEG, the log detector [26], the mean absolute amplitude and the simple square integral are several computations based on the summation of the absolute value of each sample in the EEG segment signal and, in this sense, provide other representation of the temporal characteristics of the EEG signal. The error from autoregressive (AR) modeling for different orders [25]. For each signal resulting from filtering EEG data in the classical frequency bands (δ, θ, α, β, γ), the maximum value, the standard deviation, the kurtosis and the skewness [2] are also calculated.

**Table A1:** Features extracted from the time domain.

Apply on	Features Extraction
Raw signal	Median—Mean—Variance—Root mean square amplitude—
	Difference between highest and lowest value—Skewness—
	Kurtosis—Integrated EEG—Mean absolute value—Simple
	square integral—V-order 2 and 3—Log detector—Average
	amplitude change—Difference absolute standard deviation
	value—Number of local maxima and minima—2nd and
	3rd Hjorth parameters—Zero crossing rate—Autoregressive
	modelling error (orders 1 to 9)—Non-linear energy
1st derivative	Variance—Zero crossing rate
2nd derivative	Variance—Zero crossing rate
EEG frequency bands	Maximum—Standard Deviation Value—
(δ, θ, α, β, γ)	Skewness—Kurtosis

### Appendix A.2. Frequency Domain Features

Spectral properties of the signal can be obtained by extraction of some measures on the Fourier transformed (FFT) signal. Most of these extracted measures are inspired from three studies [10,11,23]. The full list of features, extracted from the frequency domain, is listed in Table A2.

First, some features are obtained from the whole EEG power spectrum. The power of the total spectrum [26] is computed. Three spectral edge frequencies are computed as that frequencies below which 80%, 90% and 95% of the total spectral power resides [25]. Spectral moments of order 0, 1 and 2 as described in [23] are also computed. From these spectral moments, three other features are computed [23]: the power spectrum centre frequency, which is the ratio of spectral moments of order 1 to order 0; the spectral root mean square; and an index of spectral deformation based on the ratios between the spectral moments. The signal to noise ratio is obtained as the ratio of the power of the spectrum to the power of the noise which is defined as the EEG spectrum for frequencies higher than 30 Hz. Inspired from [24], the modified median frequency and the modified mean frequency are adapted to EEG signal.

δ, θ, α, β, and γ bands are directly defined from the spectrum with the same limits than those used for the time domain features. For each of these frequency band, the ratio of the area under the spectrum of the specific frequency band to the area under the whole spectrum is performed (Ratio Spectrum Area).

As in [11], we extract four other features for each of these filtered band: the non-normalized power and the log-transformation of this measure; the relative power, which is the power normalized by the total power energy; and the wavelet coefficient.

Features which provide information about change in different spectral bands are also computed: the tenth first cepstral coefficients are extracted, as suggested in [11]; we also adapt the computation of the energies, and the relative spectral differences, in the frequency bands previously described [11].

**Table A2:** Features extracted from the frequency domain.

Information about	Features Extraction
Whole spectrum	Power—Spectral Edge Frequency (80%, 90%,
	95%)—Power Spectrum Moments (orders 0, 1, 2)—
	Power Spectrum Centre Frequency—Spectral Root Mean
	Square— Index of Spectral Deformation—Signal-to-noise ratio—
	Modified Median Frequency—Modified Mean Frequency
EEG frequency bands	Ratio Spectrum Area—Non-normalized Power—
(δ, θ, α, β, γ)	Log Power—Relative Power—
	Wavelet energy (Db8 wavelet coefficients)
Changes in several	10 Cepstral Coefficients—5 Frequency-filtered band
spectral bands	energies—5 Relative Spectral Differences
